# New York Academy of Medicine

**Published:** 1888-02

**Authors:** 


### NEW YORK ACADEMY OF MEDICINE.

*A Discussion on Hernia—Treatment of Strangulated Hernia; Management Before Operation; Principles of the Operation — Conservative Treatment of Irreducible Hernia--Strangulated Hernia in Children--Telegraphic Mind-Cure.*

At the December meeting of the Academy of Medicine there was a discussion on the subject of hernia. It was opened by Dr. T. HERRING BURCHARD, who gave an exposition of the modern treatment in strangulated hernia, and urged the advantages of early operative interference. He said that, encouraged by the remarkable success obtained in the treatment of strangulated hernia by the methods suggested by Mitchell Banks, Czerny, and others, surgeons had latterly paid special attention to the radical cure by operation, as applied to cases in which strangulation existed, and the results achieved had been gratifying. The modern operation found its greatest advantage in the radical cure it secured by the complete closure of the sac and the permanent obliteration of the canal; and it had been successfully performed upon all forms of hernia--inguinal, femoral, and umbilical—with equally satisfactory results. In the light of these facts he thought that no operation for strangulated hernia could be said to be properly performed without the final closure of the inguinal canal.

With regard to the management previous to operating Dr. Burchard said that his experience had led him to the adoption of a plan of treatment substantially as follows:

1. Pain is allayed, vomiting quieted, and nervous tranquility secured by the hypodermic administration of morphia and atropia.

2. As soon as practicable a careful examination is made of the hernial tumor, any attempt at reduction being studiously avoided.

3. If evidences of inflammation are found, or if there is much swelling in the tumor, a poultice of flaxseed meal and cracked ice, or the ice-coil, is at once applied; these being dispensed with, however, if the patient is feeble or the strangulation is of long standing.

4. A stimulating enema of turpentine and oil, or a large emollient one of flaxseed tea, is administered and the rectum thoroughly washed out. If the tumor is at all distended no attempt at taxis is made until.

5. The patient is anæsthetized, permission having first been obtained to proceed with the operation in case taxis should fail.

He especially emphasized the advantages to be gained by putting the patient under full anæsthesia (preferably by chloroform), covering the tumor with a rather large and heavy ice poultice, elevating the lower extremities, and keeping the hands entirely off for a period of from thirty to forty minutes. In a number of instances, he said, he had had the satisfaction of seeing the tumor slip back of itself, almost imperceptibly, even after prolonged taxis had been unsuccessfully employed. As to just how long the attempt at reduction was justifiable, no absolute rule could be laid down. In some cases it was advisable that the trial should be kept up for twenty, or even thirty, minutes, provided always that only the most gentle manipulations should be employed.

Taxis having proved unsuccessful, the operation should at once be undertaken, and the general principles upon which, as a legitimate surgical procedure, it was based were the following:

1. A more general recognition by the profession of the value of time in the earlier stages of strangulation, so that surgical relief could generally be rendered before pathological changes which were irremediable had taken place in the tissues.

2. Modern herniotomy implies an abandonment of those uncertain and pernicious methods and practices which, founded in a false pathology, sought to relieve the strangulation by producing a condition of systemic relaxation. It is now recognized that spasm, as a causative agent in the production of strangulation, never exists.

3. Modern herniotomy restricts the employment of taxis within limits which are rational and safe. Necessary as properly directed taxis is in the reduction, by its indiscriminate and reckless use irreparable damage has not infrequently been done the intestine.

4. Modern herniotomy implies the early resort to a cutting operation. Many lives, Dr. Burchard said, had to be sacrificed before the profession seemed to realize that the gangrene and ulceration, the fœcal extravasation and stercoraceous vomiting, the peritonitis and collapse, were not necessary and integral factors of the primary condition of strangulation, but secondary complications, developing later on in the progress of the disease, and the legitimate result of delay in affording relief to the original constriction.

5. Modern herniotomy is an antiseptic operation, demanding the fullest application of Listerian principles.

6. Modern herniotomy requires in the disposition of the hernial sac the finest impartiality of judgment. Certain cases require incision, while in others the strangulation can be readily reduced without opening the sac; so that it would be folly to thus complicate the operation and expose the patient to unnecessary danger.

7. Since Mitchell Banks, of Liverpool, has urged the possibility and expediency of perfecting the old operation of kelotomy, so as to add to the operation done for the relief of strangulation the inestimable advantages of an operation for radical cure, the strongest possible encouragement has been given for the attainment of an early, thorough, and perfect operation.

Professor V. P. Gibney said that he should like to make some protest against the strong condemnation of taxis which Dr. Burchard had expressed, as he had seen a number of aggravated cases of strangulated hernia reduced under taxis as employed by the average practitioner.

Professor Robert F. Weir remarked that while the radical operation unquestionably marked an improvement in this field of surgery, it could not as yet be regarded as a perfected operation; and, having given some statistics showing the comparative frequency of relapses, he said that surgeons were everywhere practicing the operation, and he feared too freely—that is, too freely in non-strangulated hernia.

Dr. W. B. De Garmo read a paper on the conservative treatment of irreducible hernia, which did not treat of strangulated hernia, but advised a course of gentle manipulations in cases which were generally regarded as irreducible. The first thing to do, he said, was to get the mass freely movable in the sac, and then use compression, rather than ordinary taxis. In the second place, the tumor was to be separated from the vicinity of the testicle, if it was situated in the rectum. In other words, it was to be perseveringly stripped from its lower adhesions, and gradually worked upwards, no force whatever being used. The complete separation of the omentum, so frequently forming a part of the mass, was accomplished before the reduction proper was commenced. By taxis he explained that he meant the gentle manipulation of the neck of the sac, but employed in such a way as to avoid pressure back against the abdominal rings, which was apt to be resorted to in taxis as commonly practiced. The manipulations were to be kept up for from fifteen to forty-five minutes at a time and repeated once a day. In some cases he was in the habit of directing self-applied taxis. After the reduction the hernial sac usually remained, but if the hernia was kept properly in place by a well-fitting truss, the sac often disappeared in the course of time. Out of ten cases reported by Dr. De Garmo, there were seven reductions and two failures; while one case was abandoned on account of the patient's having diabetes.

An interesting part of the discussion was that in reference to strangulated hernia in children. Dr. A. G. Gerster read a paper on this subject in which he reported four cases operated upon, and said that there was one point of difference in the treatment required as compared with that in adults; this being due to the difficulty of preventing infection of the external wound in small children. Primary union did not occur after suturing, and he therefore advocated an open treatment of the wound. It was his practice to pack it with iodoform gauze in order to secure an aseptic condition.

Professor Weir said that he had had two cases in children, like those of Dr. Gerster, in which he operated; and both had proved fatal. In two other cases he had successfully reduced the hernia without operation, by resorting to the expedient of inserting the finger into the rectum, and thus combining internal with external pressure.

Professor Gibney, who has had an unusually large experience in the matter at the Hospital for Ruptured and Crippled, said that he did not recall a single instance of strangulated hernia occurring in childhood in which it was not possible to reduce the strangulation with the aid of chloroform. Furthermore, he believed that if the hernia could be reduced in children, it could be cured simply by the wearing of a suitable truss. As, in addition, the operation was a more serious one than in adults, he thought that operative interference should never be resorted to in children; and such interference became all the more unnecessary in the light of the successful results accomplished by the conservative methods advocated by Dr. De Garmo.

In concluding the discussion Dr. De Garmo said that he had met with only temporary incarceration in children. No serious results had ever occurred in his experience, and he had never found it necessary even to resort to taxis in such cases.

